# Dr. Charles S. Douglass

**Published:** 1896-10

**Authors:** 


DR. CHARLES F. DOUGLASS, graduate of the American Veterinary College, Class of 1892, died early in August in Hayti, where he had gone from his home at Kingston, Jamaica, to fill an appointment under the Haytian government.

